# Assessing environmental assets for health promotion program planning: a practical framework for health promotion practitioners

**DOI:** 10.15171/hpp.2016.19

**Published:** 2016-08-10

**Authors:** Andrew E. Springer, Alexandra E. Evans

**Affiliations:** ^1^Assistant Professor of Health Promotion and Behavioral Sciences, Michael & Susan Dell Center for Healthy Living, University of Texas Health Science Center at Houston School of Public Health, Austin, TX, USA; ^2^Associate Professor of Health Promotion and Behavioral Sciences, Michael & Susan Dell Center for Healthy Living, University of Texas Health Science Center at Houston School of Public Health, Austin, TX, USA

**Keywords:** Health needs assessment, Program planning, Health promotion, Health behavior, Environment, Ecological models

## Abstract

Conducting a health needs assessment is an important if not essential first step for health promotion planning. This paper explores how health needs assessments may be further strengthened for health promotion planning via an assessment of environmental assets rooted in the multiple environments (policy, information, social and physical environments) that shape health and behavior. Guided by a behavioral-ecological perspective- one that seeks to identify environmental assets that can influence health behavior, and an implementation science perspective- one that seeks to interweave health promotion strategies into existing environmental assets, we present a basic framework for assessing environmental assets and review examples from the literature to illustrate the incorporation of environmental assets into health program design. Health promotion practitioners and researchers implicitly identify and apply environmental assets in the design and implementation of health promotion interventions;this paper provides foundation for greater intentionality in assessing environmental assets for health promotion planning.

## Introduction

In planning health promotion interventions, conducting a *health needs assessment* is an important if not essential first step.^1-4^ While the reasons for conducting a health needs assessment vary- from identifying and prioritizing health needs and population groups most at risk as part of a community health assessment (CHA) or community health needs assessment (CHNA),^2-4^ to designing a health intervention to address a specific health problem for a given population,^1^ to other reasons such as policy development, public health assurance (e.g., enforcement of sanitary codes), and community engagement,^2,3^ central to current health needs assessment approaches has been a focus on assessing both the health needs as well as the assets or resources of a given community. This combined focus on needs and assets is reflected in current definitions of CHAs and CHNAs^2^ and reflects the *WHO Health Promotion Glossary’s* general definition of health needs assessment as “a systematic procedure for determining the nature and extent of health needs in a population, the causes and contributing factors to those needs, and the human, organizational and community resources which are available to respond to these.”^5^ The incorporation of an asset assessment holds great potential to enhance the health needs assessment process as well as program efficacy and sustainability, and as such, merits increased attention in the field of health promotion planning.

The movement toward an asset-based assessment approach was spearheaded in part by Kretzman and McKnight’s^6^ seminal work on asset mapping in the field of community organizing, which reframed community assessment from a deficit approach focused purely on needs of a community, to an approach and philosophy that promotes harnessing existing local resources and capacities for community development. An asset-based approach to community development includes identifying individual-level assets such as skills, talents and knowledge of people within a given community; organizational assets existing within the community such as community associations, local businesses, and religious organizations; organizational assets controlled from outside of the community, such as hospitals, schools and financial institutions; and physical resources such as land use.^7^ In the field of health promotion, a similar movement toward an asset-based approach took place during the same period of the 1990s, exemplified in part by a heightened focus on constructs such as *community capacity,*^8^* social capital,*^9^ and* developmental assets*.^10,11^ Currently, the incorporation of an assessment of community assets – in addition to health needs - has become a standard best practice promoted by organizations such as the National Association of County and City Health Officials (NACCHO) and the Public Health Accreditation Board in the United States.^3^

Despite the movement toward the identification of community assets in the health needs assessment process, the application of an asset assessment approach guided by theory has received little attention in the health promotion literature to date. As health behavior is at the core of disease prevention and health promotion, comprising various types of behaviors that include health promoting behaviors (e.g., physical activity and fruit and vegetable consumption), risk behaviors (e.g., substance use), self-management behaviors (e.g., asthma management), and other preventive and compliance behaviors (e.g., screening, medical visits, and medication adherence),^1^ we posit that asset assessment can be further enhanced for health promotion planning by explicitly identifying assets rooted in environments and settings that hold potential to influence the ultimate targets of health behavior and health outcomes.

With the overarching aim of contributing to the science and practice of health promotion asset assessment, the purpose of this paper is threefold: (1) to describe the rationale for balancing a needs assessment with an assessment of the assets of a priority population, their environments, and the settings that surround them; (2) to explore a basic *environmental asset* assessment framework guided by* ecological theories of health behavi*or and principles from the field of implementation science; and (3) to illustrate the application of an *environmental asset* approach within health promotion planning using examples from the scientific literature and the health promotion practice field.

## Balancing the needs assessment with an assessment of assets:

###  Why health asset assessment matters for health promotion

Several reasons exist for incorporating an assessment of assets and capacities of the priority population and their environments, including the importance of a *community empowerment vs. needs-based approach,* the opportunity to enhance* intervention effectiveness*, and the potential to increase *implementation* and *sustainability* of health promotion interventions. In the following section we explore why the incorporation of an asset assessment approach in the health needs assessment process is essential and merits heightened emphasis in CHA and intervention planning.

### Asset assessment for community empowerment

In following the credo in the medical field of “first, do no harm”, in the field of health promotion practice, we must also take caution against victimizing a priority population or community by focusing solely on the multiple problems and deficits that confront them. McKnight and Kretzman^7^ caution against the potentially adverse consequences of needs-based solutions to community development in which low-income neighborhoods may become environments of services where “…residents come to believe that their well-being depends upon being a client.” Given that all populations and communities have strengths and assets, health promotion program planners are in a unique position to co-learn and plan with communities to harness and activate these capacities and assets for health promotion planning and intervention.

### Asset assessment for enhancing intervention effectiveness

Beyond the philosophical shift of an asset assessment approach, identifying the assets and capacities in a given community holds potential to broaden our understanding of potential factors that influence and promote positive health outcomes. An asset-based approach to program planning builds from the strengths of individuals, communities and their environments and is supported by theoretical-based concepts of *positive deviance*^12^ and *resiliency,*^13^ which focus on uncovering the factors associated with “positive” health and development, and not just the factors associated with the problem. Positive social interpersonal relationships and social cohesion^14,15^ are examples of positive assets that hold potential for reducing exposure to youth risk behavior.

### Asset assessment for implementation and sustainability

An important focus of the growing field of implementation research is on understanding the context in which health interventions are delivered,^16^ which includes understanding how an intervention “couples” with the intended setting in order to increase the likelihood that programs will be effectively implemented and “stick” with the setting over time.^17,18^ Balancing the needs assessment with an assessment of a community’s assets – with specific attention to uncovering factors that can support and deliver an intervention within a given context or setting - holds potential to enhance a health program’s implementation and sustainability by allowing for the identification of potential opportunities to couple or *weave* intervention efforts into existing settings – including organizations and communities.

## Exploring a basic environmental asset assessment framework for health promotion planning

Several definitions and conceptualizations have been cited in the literature to describe the general concept of a *community asset*, which is often used synonymously with the term *resource*. In the field of community organizing, Kretzman and colleagues^19^ propose five key types of community assets: *local residents’ skills, passions, capacities, and willingness to contribute to a given project; local voluntary organizations, clubs and networks; local institutions such as schools and businesses; physical assets such as land and infrastructure;* and* economic assets*. In the context of healthy adolescent development, the Search Institute defines assets as “…important relationships, skills, opportunities and values that help guide adolescents away from risk behaviors, foster resilience, and promote thriving”, with a framework that includes both internal assets and external assets.^10^ In the field of CHNA, *The Community Tool Box*^20^ provides a robust definition of a community asset as “…anything that can be used to improve the quality of community life”, which may range from individuals, to physical structures, to community services such as public transportation.

### Applying ecological theory to asset assessment for health promotion planning

While the definitions of assets listed above share similarities, their differences underscore how asset assessment is often bound and shaped by the field for which it is being applied. In identifying assets for the specific purpose of health promotion planning and health behavior change, ecological models of health behavior provide a robust framework.^21-23^ Ecological models of behavior stem from the premise that individuals are a ‘product of their environment,’ and that environments hold the potential to directly and indirectly shape individuals’ health and health behavior,^22^ with pathways of influence that include social norms, social comparison and role models, social support, and other forms of social influence; opportunities or barriers to engage in a given behavior; information transfer; and incentive motivation via rewards or punishment.^21-27^ Central to ecological models of health behavior are the principles of: *multiple levels of influence* (e.g., societal, organizational, interpersonal and individual) and *multiple environments* (e.g., policy, information, social, physical) that shape behavior; *behavioral settings* - such as schools, workplaces, and church - which can serve to both reach populations and influence behavior; and *interactions of influence*, which refers to the interaction between and among levels or environments that may enhance or inhibit a given health outcome.^22,27^ These principles direct our attention to identifying the facets of environments and settings that can be harnessed for influencing health and health behavior.

In defining the concept of *environment*, we adhere to Albert Einstein’s broad conceptualization of environment as ‘everything that isn’t me’.^28^ In following such a broad definition, we open up the environmental space for further conceptualization and identification of assets “outside the individual” that may be harnessed toward health promotion. Although there is great potential to further conceptualize environment, we build from existing social-ecological theory^22,27^ and propose an initial framework for asset assessment guided by four key environments: *policy environment, information environment, social/organizational environment,* and* the physical environment.*

*The Policy Environment*: While it is common to separate “policy” from “environment” (e.g., “policy and environmental interventions”), we share Sallis et al’s^27^ conceptualization of policy as a representation of a specific kind of environment. By stating “policy environment”, we anchor policy to an environmental space, which may include a household setting (e.g., a family rule on TV watching), a classroom (e.g., classroom policy for earning more recess time for good behavior), a school (e.g., policy on only serving water at school events), a school district (e.g., daily PE class), or a state or nation (e.g., 30 minutes of physical activity a day for school children). Assessing existing policies and practices within different settings at the asset assessment phase allows for uncovering gaps in policy and practice, identifying policies that may adversely affect health, identifying existing policies that may not be fully implemented yet may provide an anchor for proposed health program efforts, and identifying existing policies in which a health focus can be interwoven, building off the “health in all policies” approach.^29^

*The Information Environment:* The information environment broadly refers to the messaging within a given setting that holds potential to positively or negatively influence health or health behavior. This messaging can take many forms (e.g., verbal, nonverbal, written, symbolic) and be delivered via diverse communication channels (e.g., posters, newsletter, art, social media, marketing). Numerous examples of how the information environment can influence health and behavior are provided in the literature, ranging from national campaigns such as the VERB campaign and its influence on physical activity in middle school students,^30^ to mass media campaigns for child survival,^31^ to the role of menu labeling and promotion of healthier eating,^32^ among others. As we discuss below, an important aspect of asset assessment is the exploration and identification of existing channels of communication (e.g., office newsletter, school marquee, church bulletin board) that can be incorporated into health intervention design.

*The Social, Cultural* & *Organizational Environment:* This environment broadly refers to the types of social and cultural organization that exist within a given setting (e.g., neighborhood organizations, parent-teacher associations, cultural centers, etc.) as well as the specific types of social, cultural or organizational factors that relate to health and health behavior (e.g., family meals and their effect on childhood obesity^33^; social support and physical activity in adolescents^34^). In our asset assessment phase with the CATCH Middle School Program,^35^ for example, we learned of existing social organizational activities, such as the literacy night in one school district, which then became the platform for incorporating a CATCH family health night. Asset assessment should include identifying the social organizations that exist within a given setting or community along with specific social, cultural and organizational factors and activities that can be incorporated into health promotion program planning.

*The Physical Environment*: Moos,^36^ in his work on *social ecology*, defines the physical environment as encompassing both features of the built environment (e.g., how we construct our buildings or neighborhoods) and features of the natural environment (e.g., green space). In recent years, we have seen an explosion of research on facets of the built environment in topic areas that range from physical activity,^37^ to the food environment,^38^ to mental health,^39^ among others. Beyond actual ‘space’, the built environment may also encompass aspects such as children’s access to physical activity equipment^40^ and access to outlets that sell fruits and vegetables.^41,42^ The natural environment includes access to green space- with emerging evidence that includes the positive effects of outdoors on physical activity and reduction of stress and depression.^43,44^ An assessment of the physical environment holds potential to expand intervention opportunities for a given health problem.

### Toward a conceptualization of environmental assets for health promotion

With guidance from ecological models of health behavior^21-23^ and the premise that individuals and their behavior are shaped by their environment, we propose a refined conceptualization of *community asset* rooted in the construct of *environmental asset*. The focus on *environment* aims to direct health planners to an intentional assessment of assets rooted in the multiple environments (e.g., policy, social, information and physical) and settings (e.g., home, school, workplace, and neighborhood) that hold potential to shape health and health behavior. In building from previous definitions of a community asset,^20^ we define an *environmental asset* as *any aspect of the multiple environments that surround individuals that can be harnessed toward promoting the health of individuals and populations.*

**Figure 1:**
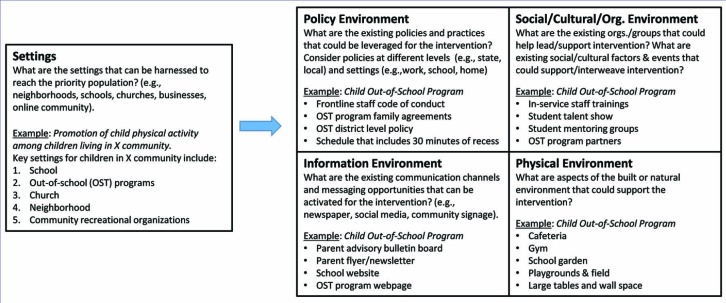
Basic environmental asset assessment framework for health promotion planning.

In Figure 1, we present a basic framework for conducting an environmental asset assessment for a health promotion and health behavior change intervention, guided by ecological models of health behavior. This framework begins with first identifying the settings where priority populations can be reached, which may include neighborhoods, schools, afterschool programs, and worksites, among other settings. The second step involves exploring the specific environmental assets within those settings that are posited to influence behavior, including *the policy environment, the information environment, the social/cultural/organizational environment,* and* the physical environment*. We offer this framework not as a recipe for environmental asset assessment, but rather as a practical and theoretical framework to complement existing health needs assessment approaches and for program planners and researchers to build from, modify, and enhance. As a brief example, we share findings in Figure 1 from a recent asset assessment workshop with afterschool program leaders (n=24) conducted as part of the Central Texas Afterschool Network BOOST Initiative, an initiative funded by the St. David’s Foundation aimed at enhancing child health in out-of-school-time programs.^45^

## Applying environmental assets to health promotion

In applying an environmental asset assessment approach to the field of health promotion, we take heart in the saying ‘old wine in new bottles’, as the general environmental asset assessment framework we propose represents a common practice health promotion practitioners and researchers have long embraced. An early and brilliant example in the field of public health of using environmental assets is the fortification of salt with iodine, in which a natural environmental asset (salt) was harnessed for delivering a health intervention (iodine) to large masses of people, and thus reducing iodine-deficiency-related disease.^46^ In the section below, we illustrate how health promotion practitioners and researchers have incorporated existing environmental assets into the design of health promotion programs and interventions. In doing so, we aim to highlight how the basic environmental asset framework described above can provide a *new bottle of wine* lens through which program planners can enhance asset assessment with greater intentionality for health promotion intervention design.

## Policy environment

- *School schedules as an environmental asset for physical activity*: In our formative work with the CATCH Middle School Project,^35^ we learned that middle school students were being dropped off at school in the morning and being directed to sit and wait in the cafeteria. Recognizing this opportunity to incorporate more physical activity opportunities, we partnered with school staff and principals to create an “Open Gym” policy/school practice in which schools opened their gyms and/or playfields for a free-play activity time with teacher supervision.
- *Campus Improvement Plans as a vehicle for child health policy*: The nonprofit organization Texas Action for Healthy Kids worked with central Texas middle schools to take advantage of the Campus Improvement Plan, a document that describes the goals, practices and activities of a given campus for enhancing the student educational experience, to interweave child health policy. Campus Improvement Plans of schools participating in the initiative resulted in increased written policy language related to coordinated school health, such as scheduling of structured activity time.^35^

## Information environment

- *Electric bills and physical activity in Brazil*. In Sao Paulo, Brazil, the Agita Sao Paulo Program delivered physical activity messaging via an existing communication channel with widespread reach: residents’ electric bills.^47^ According to the authors, this approach required no funding from the program and reached 7 million residents.
- *Soap operas and HIV prevention*. Soap operas and other entertainment media represent a powerful communication channel for interweaving health messaging, with a growing body of evidence on their effectiveness in promoting health-related knowledge, attitudes, intentions and behavior.^48^ In an episode of the soap opera *The Bold and the Beautiful*, the insertion of an HIV/AIDS subplot along with displaying a national AIDS and STD hotline resulted in dramatic increases in hotline calls.^49^
- *2-1-1 Information System and Community Health Promotion.* In the United States, the 2-1-1 system is a 3-digit phone number designated by the Federal Communications Commission as a free information resource to connect callers with health and social services in their community.^50^ An emerging body of research provides evidence on the application of this community information resource as a promising communication channel for increasing health screening and delivery of health interventions.^50-52^

## Social/cultural/organizational environment

- *Harnessing cafeteria workers for fruit and vegetable consumption*. In addition to an array of other environmental strategies, the 5-A-Day Cafeteria Power Plus project harnessed an existing school social environmental asset to positively encourage fruit and vegetable consumption in elementary school children: *cafeteria workers working on the serving line.*^53^ Verbal encouragement from cafeteria staff was found to be associated with increased child fruit and vegetable intake.^53^
- *Cultural organizations as a vehicle for healthy lifestyle promotion in Filipino-Americans*. Nutrition and physical activity were promoted via Filipino-American social clubs in San Diego, California by forming health committees and training 2-3 members of each social club in health education, behavior change skills development, and organizational policy change.^54^ The 18-month intervention resulted in significant increases in physical activity and selected dietary outcomes among study participants.^54^

## Physical environment

- *WIC clinics, farm stands and family fruit and vegetable promotion.* In order to increase access to fresh produce in central Texas, produce stands were placed at Women, Infants, and Children (WIC) clinics located in food desert type communities, which resulted in greater fruit and vegetable consumption among WIC recipients and residents living within a half mile of the farm stand.^55^
- *Schoolyards and physical activity*. In exploring opportunities to support children’s physical activity in low income communities in New Orleans, Louisiana, Farley and colleagues^56^ took advantage of an existing built environmental asset: schoolyards that were locked after school. With limited resources, schoolyards were activated for children’s afterschool play by incorporating adult supervisors and a parent permission process.^56^
- *School design and healthy eating*. A recent review by Frerichs et al^57^ provides evidence for the influence of school design on healthy eating, which includes quasi-experimental evidence that increased access to healthy items and decreased access to unhealthy items (e.g., access to healthier foods in vending machines and healthier foods on cafeteria serving lines) improves student dietary behaviors.
- *Barbershops as settings for health promotion.* A systematic review of the literature shows that beauty salons and barbershops are both feasible and effective settings for health promotion, with topics that include cancer screening, hypertension, and diabetes.^58^ Salons and barbershops are an example of a physical setting with wide reach of specific subgroups.

## Discussion

Beyond the multiple benefits of conducting a CHNA for planning interventions aimed at advancing a population’s health, conducting a CHNA has received heightened attention in recent years in countries such as the United States, where current health policy under the Patient Protection and Affordable Care Act now requires federally funded hospitals to conduct a CHNA every three years.^59^ In this paper, we provide a basic conceptual framework for enhancing the health needs assessment process for health promotion planning via an assessment of *environmental assets* (e.g., policy, information, social, and physical environment) of a given community, organization or setting. A strength of this paper is the application of ecological theory of health behavior and principles of implementation science for identifying environmental assets that hold potential to enhance the design, implementation and sustainability of interventions directed at health behavior change.

In providing the basic conceptual framework for environmental asset assessment described in this paper, we recognize that there are other promising frameworks that hold value in guiding asset assessment for different fields and different purposes. For example, Green andHaines^60^ conceptualize asset assessment for community development in terms of seven types of capital: human, social, physical, financial, environmental, political and cultural capital. As we describe in this paper, we posit that asset assessment can be enhanced via the lens of the field for which it is being applied. The basic environmental asset framework proposed in this paper for the field of health promotion planning differs from others by specifically applying both a behavioral-ecological perspective- one that seeks to identify environmental assets that can shape behavior, and an implementation science perspective- one that seeks to ‘couple’ and ‘interweave’ health promotion intervention strategies into existing environmental assets. This approach aims to increase health promotion intervention effectiveness while increasing implementation and sustainability of health promotion intervention initiatives.

Although we describe in this paper various examples of how environmental assets have been applied in health promotion research and practice, we recognize that the conceptual framework proposed here is basic, theory-based, and merits further empirical evaluation. This limitation notwithstanding, we hope that this paper serves as a catalyst to continue to grow the science and practice around environmental asset assessment for health promotion planning.

## Conclusion and future directions

While many health promotion practitioners and researchers implicitly identify and apply environmental assets in the design and implementation of health promotion interventions, this paper provides a foundation for greater intentionality in assessing *environmental assets* that hold potential to directly shape health and health behavior. As described herein, the concept of environmental asset assessment holds great potential for furthering the field of health needs assessment. Future directions of this work include the development of a *common vocabulary and constructs*, further conceptualization of additional ‘environments’ important for health promotion- such as the arts and aesthetic environment (see Semenza and Krishnasamy^61^ for inspiring examples), identification of *methods*, and attention to *process* for conducting an environmental asset assessment.

## Acknowledgements

We gratefully acknowledge Dr. Chris Markham and Dr. Kay Bartholomew, lead editors and authors of Planning health promotion programs: An Intervention Mapping Approach, who provided initial encouragement for this paper. We also express our gratitude to Dr. Cheryl Perry and Dr. Chris Markham from the Department of Health Promotion and Behavioral Sciences at the University of Texas Health Science Center at Houston (UTHealth) School of Public Health for their insightful and constructive comments of earlier drafts. Lastly, we greatly value the editorial and research support provided by Ms. Sarah Bentley, Graduate Research Assistant, and Mr. Tim Cooley, Dell Undergraduate Scholar, at the Michael & Susan Dell Center for Healthy Living at the University of Texas School of Public Health-Austin Regional Campus.

## Funding

This paper was supported in part by a community health grant from the Michael & Susan Dell Foundation, which provides funding for the Michael & Susan Dell Center for Healthy Living/UTHealth School of Public Health-Austin where the authors are based.

## Ethics approval

This paper is based primarily on a review of existing literature and does not report any individual data. The example cited on our work with the CTAN BOOST Initiative is based on study protocols for that project that were reviewed and approved by the University of Texas Health Science Center Committee for the Protection of Human Subjects (IRB #HSC-SPH-13-0190).

## Competing interests

The authors formally declare that we have no financial interest in the research or conflict of interest.

## Authors’ contributions

AS and AE jointly conceived this paper and prepared and edited the manuscript.

## References

[1] Bartholomew Eldredge LK, Markham CM, Ruiter RA, Fernández ME, Kok G, Parcel GS, editors. Planning health promotion programs: An Intervention Mapping approach. 4th ed. San Francisco, CA: Jossey-Bass; 2016.

[2] U.S. Centers for Disease Control and Prevention. Community Health Assessment for Population Health Improvement: Resource of Most Frequently Recommended Health Outcomes and Determinants. Atlanta, GA: Office of Surveillance, Epidemiology, and Laboratory Services; 2013.

[3] National Association of County and City Health Officials (NACCHO). Community Health Assessment and Improvement Planning. http://www.naccho.org/programs/public-health-infrastructure/community-health-assessment. Accessed 17 July 2016.

[4] CDC Community Health Improvement Navigator. Assess Needs and Resources. http://www.cdc.gov/chinav/tools/assess.html. Accessed 29 June 2016.

[5] Smith BJ, Tang KC, Nutbeam D (2006). WHO health promotion glossary: new terms. Health Promot Int.

[6] Kretzman JP, McKnight JL. Building Communities from the Inside Out: A Path Toward Finding and Mobilizing a Community’s Assets. Evanston, IL: Institute for Policy Research; 1993.

[7] McKnight JL, Kretzman JP. Mapping Community Capacity. Evanston, Il: Institute for Policy Research; 1996.

[8] Goodman RM, Speers MA, McLeroy K, Fawcett S, Kegler M, Parker E (1998). Identifying and defining the dimensions of community capacity to provide a basis for measurement. Health Educ Behav.

[9] Lochner K, Kawachi I, Kennedy BP (1999). Social capital: a guide to its measurement. Health Place.

[10] Scales PC, Leffert N. Developmental assets: A synthesis of the scientific research on adolescent development. 2nd ed. Minneapolis, MI: Search Institute; 2004.

[11] Catalano RF, Berglund ML, Ryan JA, Lonczak HS, Hawkins JD (2002). Positive youth development in the united states: research findings on evaluations of positive youth development programs. Prevention & Treatment.

[12] Wishek SM, Van der Vynckt S (1976). The use of nutritional “positive deviants” to identify approaches for modification of dietary practices. Am J Public Health.

[13] Zimmerman MA (2013). Resiliency theory: a strengths-based approach to research and practice for adolescent health. Health Educ Behav.

[14] Evans AE, Sanderson M, Griffin S, Reininger B, Vincent ML, Parra-Medina D, et al. An exploration of the relationship between youth assets and engagement in risky sexual behaviors. J Adolesc Health 2004;35:424.e21-30. doi: 10.1016/j.jadohealth.2004.02.008. 15488436

[15] Springer AE, Cuevas Jaramillo MC, Ortiz Y, Case K, Wilkinson A. School social cohesion, student-school connectedness and bullying in Colombian adolescents. Glob Health Promot. 2015. pii: 1757975915576305. 10.1177/1757975915576305 25878143

[16] Neta G, Glasgow RE, Carpenter CR, Grimshaw JM, Rabin BA, Fernandez ME (2015). A framework for enhancing the value of research for dissemination and implementation. Am J Public Health.

[17] Hawe P, Shiell A, Riley T (2009). Theorising interventions as events in systems. Am J Community Psychol.

[18] Saunders RP, Evans AE, Kenison K, Worman L, Dowda M, Chu YH (2013). Conceptualizing, implementing, and monitoring a structural health promotion intervention in an organizational setting. Health Promot Pract.

[19] Kretzman JP, McKnight JL, Dobrowolski S, Puntenney D. Discovering community power: a guide to mobilizing local assets and your organization’s capacity. Northwestern University: Asset-Based Community Development Institute School of Education and Social Policy; 2005. http://www.abcdinstitute.org/docs/kelloggabcd.pdf. Accessed 30 June 2016.

[20] The Community Tool Box. Section 8. Identifying Community Assets and Resources. Work Group for Community Health and Development, University of Kansas.http://ctb.ku.edu/en/table-of-contents/assessment/assessing-community-needs-and-resources/identify-community-assets/main. Accessed 30 June 2016.

[21] Bronfenbrenner U (1986). Ecology of the family as a context for human development: research perspectives. Dev Psych.

[22] Sallis JF, Owen N, Fisher EB. Ecological models of health behavior. In: Glanz K, Rimer B, Viswanath K, eds. Health Behavior and Health Education. 4th ed. San Francisco, CA: Jossey-Bass; 2008. p. 465-82.

[23] Hovell MF, Wahlgren DR, Adams M. The logical and empirical basis for the Behavioral Ecological Model. In: DiClemente RJ, Crosby R, Kegler M, eds. Emerging theories and models in health promotion research and practice: Strategies for enhancing public health. 2nd ed. San Francisco: Jossey-Bass; 2009. p. 415-50.

[24] Perry CL, Jessor R (1985). The concept of health promotion and the prevention of adolescent drug abuse. Health Educ Q.

[25] McAlister AL, Perry CL, Parcel G. How individuals, environments and health behaviors interact. In: Glanz K, Rimer BK, Viswanath K, eds. Health Behavior and Health Education: Theory, Research and Practice. 4th ed. San Francisco, CA: John Wiley & Sons; 2008. p. 169-88.

[26] Maibach EW, Abroms LC, Marosit M (2007). Communication and marketing as tools to cultivate the public’s health: a proposed “people and places” framework. BMC Public Health.

[27] Sallis JF, Cervero RB, Ascher W, Henderson KA, Kraft MK, Kerr J (2006). An ecological approach to creating active living communities. Annu Rev Public Health.

[28] Albert Einstein Quotes. BrainyQuote website. http://www.brainyquote.com/quotes/quotes/a/alberteins165189.html.Accessed 17 July 2016.

[29] Rudolph L, Caplan J, Ben-Moshe K, Dillon L. Health in All Policies: A Guide for State and Local Governments. Washington, DC and Oakland, CA: American Public Health Association and Public Health Institute; 2013.

[30] Huhman ME, Potter LD, Nolin MJ (2010). The Influence of the VERB Campaign on Children’s Physical Activity in 2002 to 2006. Am J Public Health.

[31] Naugle DA, Hornik RC (2014). Systematic review of the effectiveness of mass media interventions for child survival in low- and middle-income countries. J Health Commun.

[32] Sinclair SE, Cooper M, Mansfield ED (2014). The Influence of menu labeling on calories selected or consumed: a systematic review and meta-analysis. J Acad Nutr Diet.

[33] Berge JM, Wall M, Hsueh TF, Fulkerson JA, Larson N, Neumark-Sztainer D (2015). The protective role of family meals for youth obesity: 10-year longitudinal associations. J Pediatr.

[34] Mendonça G, Cheng LA, Mélo EN, Farias Junior JC (2014). Physical activity and social support in adolescents: a systematic review. Health Educ Res.

[35] Springer AE, Kelder SH, Byrd-Williams CE, Pasch K, Ranjit N, Delk J (2013). Promoting energy-balance behaviors among ethnically diverse adolescents: overview & baseline findings of the Central Texas CATCH Middle School Project. Health Educ Behav.

[36] Moos RH. Social-Ecological Perspectives on Health. In: Stone GC, Cohen F, Adler NE, eds. Health Psychology: A Handbook. San Francisco, CA: Jossey-Bass; 1980.

[37] Bancroft C, Joshi S, Rundle A, Hutson M, Chong C, Weiss CC (2015). Association of proximity and density of parks and objectively measured physicalactivity in the United States: a systematic review. Soc Sci Med.

[38] Eyler AA, Blanck HM, Gittelsohn J, Karpyn A, McKenzie TL, Partington S (2015). Physicalactivity and food environment assessments: implications for practice. Am J Prev Med.

[39] Francis J, Wood LJ, Knuiman M, Giles-Corti B (2012). Quality or quantity? Exploring the relationship between Public Open Space attributes and mental health in Perth, Western Australia. Soc Sci Med.

[40] Verstraete SJ, Cardon GM, De Clercq DL, De Bourdeaudhuij IM (2006). Increasing children’s physical activity levels during recess periods in elementary schools: the effects of providing game equipment. Eur J Public Health.

[41] Evans A, Banks K, Jennings R, Nehme E, Nemec C, Sharma S (2015). Increasing access to healthful foods: a qualitative study with residents of low-income communities. Int J Beh Nutr Phys Act.

[42] Dave J, Evans AE, Saunders RP, Watkins K, Pfeiffer K (2009). Correlates of availability and accessibility of fruit and vegetables in homes of low-income Hispanic families. Health Educ Res.

[43] Largo-Wight E (2011). Cultivating healthy places and communities: evidence-based nature contact recommendations. Int J Environ Health Res.

[44] Lee AC, Maheswaran R (2011). The health benefits of urban green spaces: a review of the evidence. J Public Health.

[45] Central Texas Afterschool Network (CTAN). CTAN BOOST Initiative. http://ctanafterschool.com/BOOST/.Accessed 29 June 2016.

[46] Delange F, de Benoist B, Pretell E, Dunn JT (2011). Iodine deficiency in the world: where do we stand at the turn of the century?. Thyroid.

[47] Matsudo V, Matsudo S, Andrade D, Araujo J, Andrade E, de Oliveira LC (2002). Promotion of physical activity in a developing country: the Agita São Paulo experience. Public Health Nutr.

[48] Shen F, Han JA (2014). Effectiveness of entertainment education in communicating health information: a systematic review. Asian J Commun.

[49] Kennedy MG, O’Leary A, Beck V, Pollard K, Simpson P (2004). Increases in Calls to the CDC National STD and AIDS Hotline Following AIDS-Related Episodes in a Soap Opera. J Commun.

[50] Hall KL, Stipelman BA, Eddens KS, Kreuter MW, Bame SI, Meissner HI (2012). Advancing collaborative research with 2-1-1 to reduce health disparities: challenges, opportunities and recommendations. Am J Prev Med.

[51] Kreuter MW, Eddens KS, Alcaraz KI, Rath S, Lai C, Caito N (2012). Use of cancer control referrals by 2-1-1 callers: a randomized trial. Am J Prev Med.

[52] Roux AM, Herrera P, Wold CM, Dunkle MC, Glascoe FP, Shattuck PT (2012). Developmental and autism screening through 2-1-1: reaching underserved families. Am J Prev Med.

[53] Perry CL, Bishop DB, Taylor GL, Davis M, Story M, Gray C (2004). A randomized school trial of environmental strategies to encourage fruit and vegetable consumption among children. Health Educ Behav.

[54] Dirige OV, Carlson JA, Alcaraz J, Moy KL, Rock CL, Oades R (2013). Siglang Buhay: nutrition and physical activity promotion in Filipino-Americans through community organizations. J Public Health Manag Pract.

[55] Evans A, Jennings R, Smiley A, Medina JL, Sharma SV, Rutledge R (2012). Introduction of farm stands in low-income communities increases fruit and vegetable consumption among community residents. Health Place.

[56] Farley TA, Meriwether RA, Baker ET, Watkins LT, Johnson CC, Webber LS (2007). Safe play spaces to promote physical activity in inner-city children: results from a pilot study of an environmental intervention. Am J Public Health.

[57] Frerichs L, Brittin J, Sorenson D, Trowbridge MJ, Yaroch AL, Siahpush M (2015). Influence of school architecture and design on healthy eating: a review of the evidence. Am J Public Health.

[58] Linnan LA, D’Angelo H, Harrington CB (2014). A literature synthesis of health promotion research in salons and barbershops. Am J Prev Med.

[59] Barnett K. Best Practices for Community Health Needs Assessment and Implementation Strategy Development: A Review of Scientific Methods, Current Practices, and Future Potential. Report of Proceedings from a Public Forum and Interviews of Experts; 2012. http://www.phi.org/uploads/application/files/dz9vh55o3bb2x56l crzyel83fwfu3mvu24oqqvn5z6qaeiw2u4.pdf. Accessed 30 June 2016.

[60] Green GP, Haines AL. Asset Building & Community Development. 4th ed. Los Angeles, CA: Sage Publications; 2016.

[61] Semenza JC, Krishnasamy PV (2007). Design of a health-promoting neighborhood intervention. Health Promot Pract.

